# Free flow electrophoresis allows quick and reproducible preparation of extracellular vesicles from conditioned cell culture media

**DOI:** 10.20517/evcna.2021.26

**Published:** 2022-03-16

**Authors:** Simon Staubach, Tobias Tertel, Bernd Walkenfort, Dominik Buschmann, Michael W. Pfaffl, Gerhard Weber, Bernd Giebel

**Affiliations:** ^1^Institute for Transfusion Medicine, University Hospital Essen, University of Duisburg-Essen, Essen 45147, Germany.; ^2^Imaging Center Essen (IMCES) Electron Microscopy Unit, University Hospital Essen, University of Duisburg-Essen, Essen 45147, Germany.; ^3^Institute of Animal Physiology and Immunology, Technical University of Munich, Freising 85354, Germany.; ^4^FFE Service GmbH, Feldkirchen 85622, Germany.

**Keywords:** Extracellular vesicles, exosomes, mesenchymal stem cells, mesenchymal stromal cells MSCs, MSC-EVs, free-flow electrophoresis

## Abstract

**Aim:**

Despite intensive research during the last decade, it remains challenging to prepare extracellular vesicles (EVs) of high purity, especially from primary body liquids or protein-rich conditioned media. For now, time-consuming combinations of at least two orthogonal methods, e.g., density and size separation, are required to enrich EVs to high purity, often at the expense of processing time. Therefore, novel technologies are required that allow EV preparation in acceptable time intervals and to fair purities. Free-flow electrophoresis (FFE) constitutes a well-established semi-preparative method to separate and prepare analytes, e.g., by inherent differences in their electric charges. FFE combines a flow-driven longitudinal transport of sample material with vertical electrophoresis and allows the separation of sample components into up to 96 different fractions. It was our aim to evaluate the potential of FFE for the separation of EVs from other sample components of EV-containing protein-rich conditioned cell culture media.

**Methods:**

Exemplarily, conditioned media of mesenchymal stem/stromal cells raised in the presence of EV-containing 10% human platelet lysate were processed. We analyzed the obtained fractions by different technologies, including imaging flow cytometry, western blot and nanoparticle tracking analysis.

**Results:**

We demonstrate that FFE quickly and reproducibly separates EVs from a huge proportion of molecules included in the original sample.

**Conclusion:**

Our results qualify FFE as a feasible, quick and reproducible technology for the preparation of *bona fide* EVs.

## INTRODUCTION

Virtually all cells release different types of membrane-surrounded nano- and micron-sized particles into their extracellular environment. Depending on their subcellular origin, these extracellular vesicles (EVs) are discriminated into different subtypes^[1]^. The most prominent EV types are exosomes (70-150 nm) arising from the endosomal system, microvesicles (100-1000 nm) budding from the plasma membrane, and apoptotic bodies, membrane-surrounded large fragments of dying cells (up to several micrometers)^[2]^. Apparently, EVs are assembled in cell type specific manners, and a proportion of them mediates complex interactions at local and distant sites in both healthy and pathological conditions^[3]^. To unravel their functions, it is a common strategy to prepare EVs and analyze their molecular content in larger detail, e.g., by proteome or RNA profiling. Traditionally, differential centrifugation-based methods are used for the enrichment of small (exosome-)sized EVs^[4]^. More recently, size exclusion technologies have become popular, originally introduced for EV preparation many years ago^[5-7]^. Lipoproteins and protein aggregates are difficult to remove with any of the given technologies, particularly when it comes to primary body liquids, serum- or human platelet lysate (hPL)-containing media. To obtain relatively pure EV samples, the method of density gradient centrifugation has been combined with size exclusion chromatography to separate EVs from most of the lipoproteins and protein aggregates^[8,9]^. Despite the high purity of the obtained EVs, the recovery is relatively low and the whole procedure is very time-consuming. Thus, the EV field urgently requires novel methods allowing preparation of EVs with improved purities and recoveries in a quick and reproducible manner.

**Figure 1:**
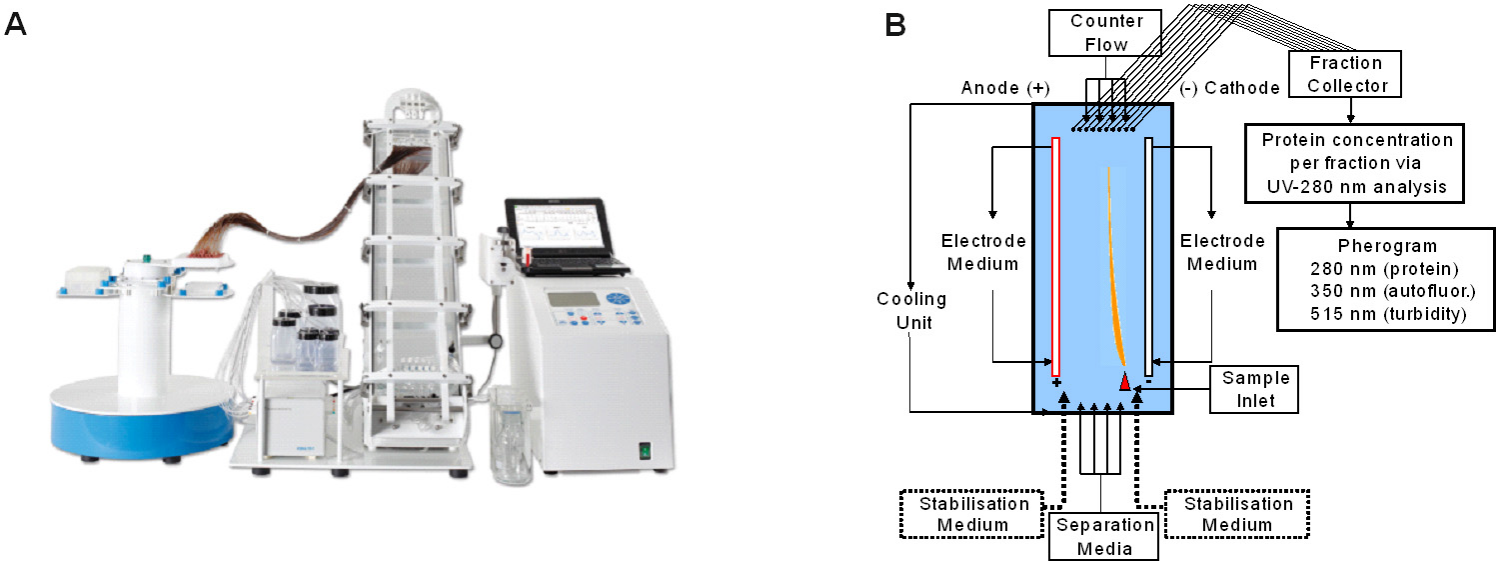
Free flow electrophoresis (FEE) and its principle. (A) Image of the FFE device developed by FFE Service. (B) Principle of the FFE device, as described in detail in the text: analytes loaded into the sample inlet are transported by a longitudinal flow and separated in a vertical electric field (see orange line as an example for one analyte). The migration speed of given analytes within the electric field is related to their isoelectric points and depends on the pH values of separation buffers within the separation area. To avoid analytes getting into contact with either electrode, specific stabilization buffers that regularly feature higher pH values protect the electrodes. At the end of the separation chamber driven by a counterflow, separated analytes are collected in 96 different pores connected with hoses to a fraction collector loading samples into 96-well plates. By spectral analyses at different wave lengths, pherograms of the fractioned samples are recorded.

Free-flow electrophoresis (FFE) is a matrix-free, well-established method for the separation of a wide variety of charged or chargeable analytes [Figure 1A]. It has been used successfully for the separation and preparation of cells; proteins in cell lysates and plasma; enzymes from extracts of bacteria, micro-organisms and mammalian cell line cells; and the preparation of cellular organelles such as peroxisomes^[10-13]^. The central unit of FFE is a separation chamber, mainly composed of a separation plate and a 0.2 mm distanced front plate including a longitudinally arranged anode and cathode [Figure 1B]. Following assembly, buffers with defined pH values are loaded into the vertically arranged separation buffer inlets at the lower edge of the separation chamber. The buffers are continuously transported along the longitudinal axis of the separation chamber by a constant laminar flow, forming concrete longitudinal buffer lanes. Likewise, the sample to be separated is applied at a concrete vertical position at the front end of the horizontal lane and transported by the laminar flow together with the respective buffer along the longitudinal axis [Figure 1]. Typically, buffer application schemes are designed such that the buffer with the lowest pH is closest to the anode and that pH values of the buffers gradually increase towards the cathode. Driven by a vertical electric field and depending on their electric charge, sample components migrate vertically through different buffer zones. The migration speed and distance of each sample component depend on its charge density and/or isoelectric point (pI). Components with higher negative charge densities or lower pIs migrate more quickly towards the anode than those with lower charge densities and higher pIs. Thus, the higher is the charge density or the lower is the pI, the quicker the analytes approach the anode. Still being transported by the horizontal laminar flow, separated sample components continuously migrate towards the top of the separation chamber where they are collected by a collector unit in up to 96 different vertically arranged fractions. For the initial analysis, obtained fractions are usually analyzed in a microtiter plate reader for their light absorption and emission capabilities at different wavelengths. Absorption at 280 nm reflects the protein contents of the fraction, emission at 350 nm the autofluorescence of ingredients illuminated at 280 nm, and absorption at 515 nm the turbidity caused by solid ingredients such as protein aggregates, EVs, and other particles and solid compounds.

Aiming to assess the feasibility and reproducibility of FFE for the preparation of EVs from complex fluids, we decided to use 48 h conditioned media (CM) from human bone marrow-derived MSCs grown in 10% hPL supplemented, non-EV-depleted media. Coupled with our interest in translating MSC-EVs into the clinics^[14,15]^, we routinely characterize obtained EV preparations by applying standard EV characterization technologies as recommended by the *minimal information for studies of extracellular vesicles* 2018 (MISEV2018) guidelines^[16]^. Furthermore, we previously optimized imaging flow cytometry (IFCM) protocols for the characterization of EVs at the single-EV level and have started to analyze EV contents in different biofluids including EV preparations obtained with different protocols by IFCM in addition^[17-21]^.

Here, using as an example one of our standard MSC-EV preparations^[14,22]^ and MSC-CMs, we developed and optimized an FFE protocol with separation buffers of different pH values for the reproducible preparation of respective EVs.

## METHODS

### Preparation of MSC-CMs

MSCs were raised from samples of healthy bone marrow donors following informed consent according to the Declaration of Helsinki, exactly as described before^[14,23]^. Their usage was approved by the ethics committee of the University of Duisburg-Essen (12-5295-BO). Briefly, obtained MSCs were expanded in DMEM low glucose (PAN Biotech, Aidenbach, Germany) supplemented with 10% hPL^[24,25]^, 100 U/mL penicillin-streptomycin-glutamine (Thermo Fisher Scientific, Darmstadt, Germany), and 5 IU/mL heparin (Ratiopharm, Ulm, Germany) at 37 °C in a humidified 5% CO_2_ atmosphere. Upon reaching 50% confluency, CMs were exchanged every 48 h until MSCs reached a density of 80%-90% confluency. After harvesting, CMs were spun at 2000 *g* for 15 min in a 5810R centrifuge (rotor A-4-81, Eppendorf, Hamburg, Germany) to remove residual cells and larger particles. Thereafter, CMs were stored at -20 °C until usage. MSC characteristics and absence of mycoplasma infections were documented in regular intervals, exactly as described before^[14,23]^. The MSC-CMs used in this study were obtained from approximately 6 × 10^6^ cells. For one of the MSCs, we used 24 h medium exchange intervals for all other 48 h intervals.

### Preparation of MSC-EVs

The MSC-EV preparation used in this study (MSC-EVs 31.2) was comprehensively characterized before, and it has been tested for its therapeutic activity in an ischemic stroke model^[26]^. Briefly, MSC-EVs 31.2 were prepared from 48 h CM of 4.3 × 10^8^ cells (4.5 L MSC-CM) applying our reported PEG/UC procedure^[14,22]^. The EV preparation was characterized by nanoparticle tracking analysis (NTA) and Western blot. The average particle sizes were 108.2 nm; the particle concentration was 2.8 × 10^11^ particles per mL; and the protein concentration was 7.7 mg protein/mL. The following administration into ischemic stroke mice, samples of this MSC-EV preparation mediated neuroprotective effects^[26]^.

### Free flow electrophoresis

FFE experiments were performed on FFE NextGen systems (FFE Service GmbH, Feldkirchen, Germany), equipped with nine inlets for the loading of the separation buffers at the front and three inlets for the counterflow buffers at the end of the separation chamber. All separations were performed at 10 °C, and 500 mm × 100 mm separation chambers were used with 0.2 mm gap width, covered with transparent plastic film.

The following FFE workflow was used [Supplementary Figure 1]. Before loading the separation chambers with the different buffers air bubble-free, the appropriate assembly of the FFE device was confirmed by running a routine program checking for the tightness of the device and the homogeneity of the laminar fluid stream within the whole chamber. To this end, a so-called stripe test was performed, in which the nine inlets at the front of the separation chamber were fed alternating with clear and pink colored water. Without applying an electric field, it was proven visually and by spectral analysis that, under laminar flow, aqueous lanes remain straight without intermingling with their neighbor lanes [Supplementary Figure 2A]. Next, the separation chamber was loaded with the separation buffers of choice flanked by the anode and cathode stabilization buffers as provided below. Depending on the detailed separation protocol, variable areas of the separation chamber were chosen for the sample fractioning, i.e., by selecting the numbers of buffer inlets to be filled with separation buffers at the front of the separation chamber. To control the electrophoretic separation performance, a mixture of different colored dyes with different pIs (range of pI 4-8) was administered into the loading inlet, applying a laminar flow of 201 mL/h and an electric voltage of 1000 V regularly, resulting in a current of approximately 190 mA [Supplementary Figure 2B]. The accuracy of the electrophoresis-driven separation process was controlled visually. Following appropriate separation of the colored dye mix, the EV-containing samples were loaded into specific sample inlets with a flow of 7.5 mL/h. Simultaneously, the laminar flow samples were separated by electrophoresis (1000 V, ~190 mA). Upon applying a counterflow at the end of the separation chamber, typically with a flow rate of 195 mL/h, sample fractions were collected via a collection unit installed at the end of the separation chamber allowing the collection of up to 96 different fractions in 96-well microtiter plates, typically as 150 μL aliquots per well. Depending on the protocol and continuous sample loading, separation time and the corresponding volume of each obtained fraction can be increased. Within 27 min, up to approximately 1.8 mL per sample fraction can be obtained, which in our experimental series were collected in 96-well polypropylene deep-well plates with loading capacities of 2 mL per well (polypropylene, Protein LoBind, Eppendorf, Hamburg, Germany). Typically, if larger (scaled) sample fractions are collected in deep-well plates, conventional microtiter plates are filled with 150 μL aliquots per sample fraction immediately before (pre-scaled sampling) and after loading of the deep well plates (post-scaled sampling). Analyses of the fractions collected in the microtiter plates before and following the scaled sampling retrospectively allow evaluation of the stability of the separation process.

Spectroscopic analyses of the collected sample fractions, typically in microtiter plates, for each of the fractioned samples were performed in a microplate reader (Tecan M200, Tecan, Männedorf, Switzerland) equipped with UV-Vis and fluorescence spectroscopy detectors and I-control 1.8 software (Tecan). Briefly, the protein content of the sample fractions was analyzed by absorption of UV light at 280 nm excitation, the autofluorescence of 280 nm illuminated fractions at 350 nm emission, and their turbidity by absorption at 515 nm extinction; 10 nm bandwidth and a photomultiplier gain setting of 80 were used. The light absorbance values of the different sample fractions were plotted in pherograms across all sample fractions. Deep-well plates loaded with up to 96 different sample fractions were stored at -80 °C until further processing. pH analyses of the collected samples were performed in an automatized manner with a Tecan MSP9259 microplate robotic system (Tecan) equipped with a WTW inoLab pH730 pH-meter (Weilheim, Germany) and “gemini for miniPrep” software.

**Figure 2:**
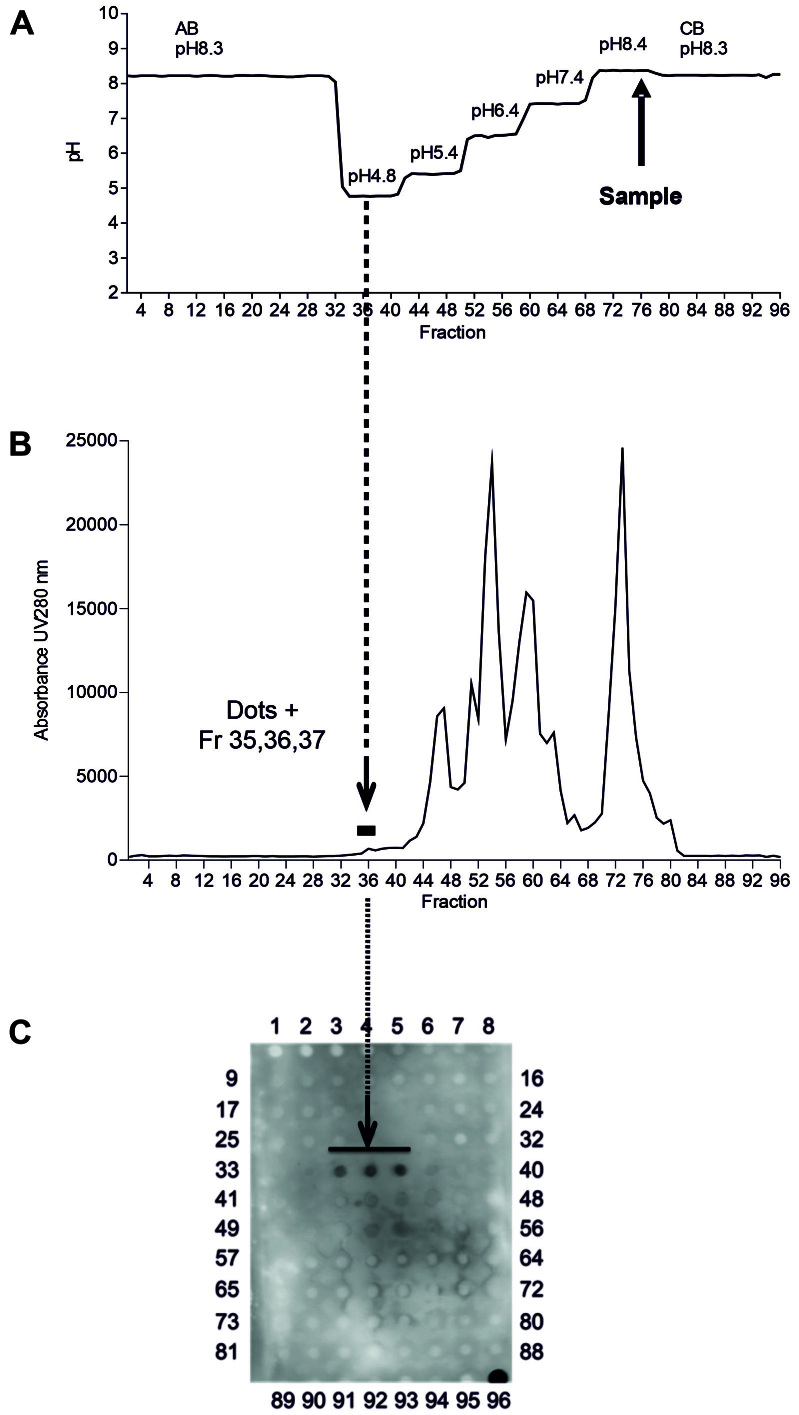
Free flow electrophoresis (FEE) effectively separates components of MSC-EV preparations. (A) A five-step pH-profile (pH 4.8, 5.4, 6.4, 7.4, and 8.4) was used for the fractioning of a 133 μL sample of a well-characterized MSC-EV preparation that was applied at the vertical position corresponding to that of the collected Fraction 78. An interval zone electrophoresis was performed at 1000 V for 6 min in a 6 cm broad separation area. (B) A pherogram of the protein content within the different fractions. (C) The results of dot blot analysis of all 96 obtained fractions using a mixture of anti-CD9 (VJ1) and anti-Syntenin (EPR8102) antibodies. As a control, at Position 96, 15 μg of a PEG precipitated MSC-EV sample were applied. Fractions delivering positive dot blot signals (Fractions 35-37) are labeled by arrows and the black line in (C).

For the initial experiments, we used an MSC-EV preparation that had been comprehensively characterized before^[26]^. For the fractioning of this sample, an interval zone electrophoresis protocol was used (longitudinal transport occurs before and after but not during electrophoresis), and the FFE separation chamber was loaded via five inlets with five separation buffers (10 mM Tris-acetate) of different pH values (pH 4.8, 5.4, 6.4, 7.4, and 8.4). The electrode stabilization buffer, 150 mM Tris-acetate pH 8.3, was loaded into two inlets in juxtaposition of the anode and one inlet to the cathode [Figure 2A]. The counterflow buffer was a 250 mM mannitol solution. The sample fractioning time was adjusted to 6 min with constant electrophoresis.

**Figure 3:**
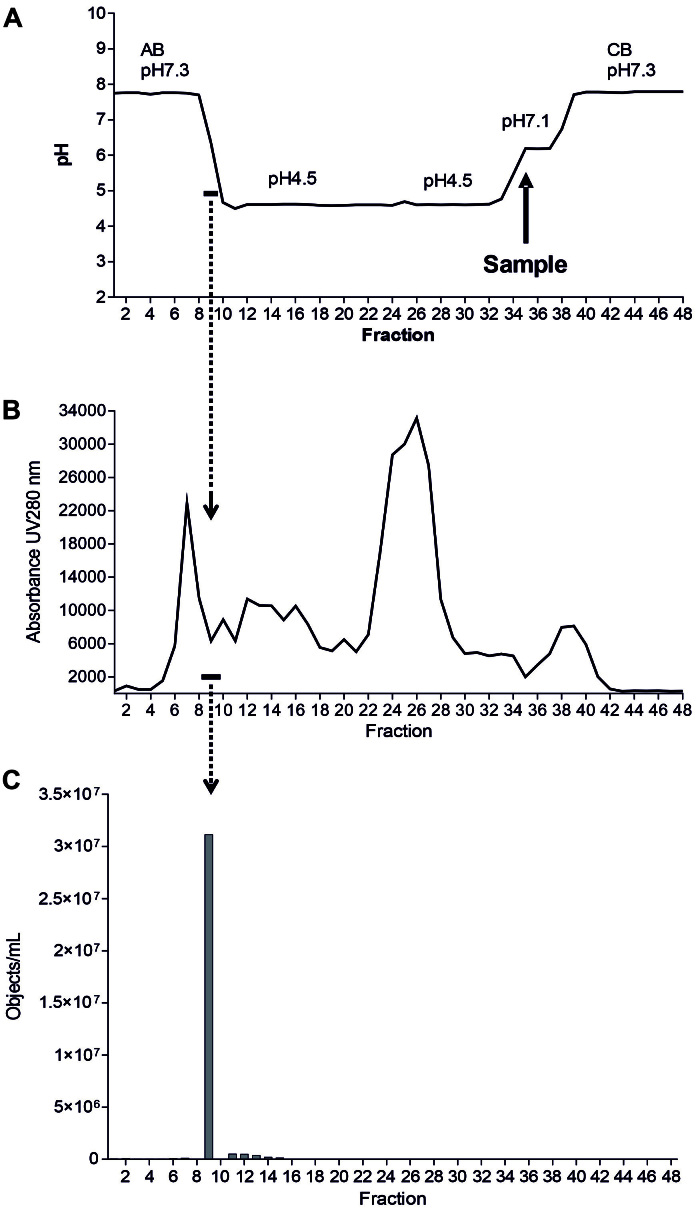
Imaging flow cytometry (IFCM) recovers CD9^+^ objects mainly in one free flow electrophoresis (FEE) fraction. (A) Following optimization, a three-step pH profile (pH 4.5, 4.5, and 7.1) was set up for the fractioning of 3.125 mL of a given MSC-CM. The sample was applied at the vertical position corresponding to that of the collected Fraction 35. The FFE process of continuous zone electrophoresis was performed at 1000 V for 3.5 min in a 3 cm broad separation area. (B) A pherogram of the protein content within the different fractions. (C) The results of IFCM analyses of 25 μL aliquots of obtained fractions that had been stained with anti-CD9 antibodies. The fraction containing detectable CD9^+^ objects (Fraction 9) is labeled by arrows and the black line in (B).

For the separation of the MSC-CMs a continuous zone electrophoresis process was used (longitudinal transport and electrophoresis occur simultaneously). Here, three separation buffers were loaded via three separate loading inlets. Buffer 1 was 15 mM Tris-HCl adjusted with e-aminocaproic acid (EACA) to pH 4.5; Buffer 2 was 5 mM Tris, titrated with acetic acid to pH 4.5, and Buffer 3 was 15 mM Tris, titrated with acetic acid to pH 6.0. The separation buffers were flanked by the anode and cathode stabilization buffers (170 mM Tris, 130 mM acetic acid, pH 7.3) [Figure 3A]. In addition, 300 mM bisamino-trismethan (BisTRIS) was used as a counterflow buffer applied via the three counterflow buffer inlets at the top end of the separation chamber.

### Dot blot

Optitran Whatman BA-S83/0.45 μm nitrocellulose membranes (Whatman GmbH, Dassel, Germany) were cut and placed into the Dot blot chamber (96-well Bio-Dot®, BioRad, Feldkirchen, Germany). Then, 200 μL of each FFE separated fraction were transferred to 1 of the 96 wells of the Dot blot chamber. Next, 15 μg of an unfractioned PEG/UC MSC-EV preparation were diluted to final volumes of 200 μL and applied as positive control. Applying a water jet vacuum pump, liquid phases of the loaded samples were sucked through the membrane. Thereafter, the blot aperture was disassembled and the membrane air-dried for 1-2 min. Subsequently, the membrane was blocked under low agitation in 5% skim milk powder solution (TBST, Sigma Aldrich, Taufkirchen, Germany) for 30 min at room temperature. To label exosomal antigens, mouse anti-CD9 antibodies (VJ1, 1:1000, kindly provided by Francisco Sánchez-Madrid) and rabbit anti-Syntenin antibodies (EPR8102, 1:1000, 1 h, RT, Abcam, Cambridge, United Kingdom, ab133267) were applied in TBS-0.1% Tween-20 (TBS-T) containing 5% (w/v) skim milk powder (Sigma-Aldrich) for 1 h at room temperature. Following incubation, membranes were rinsed and washed three times for 5 min and once for 10 min in TBS-T. For the detection, membranes were incubated with peroxidase AffiniPure F(ab')2 fragment donkey anti-mouse IgG (1:10,000, polyclonal 715-036-150; Jackson ImmunoResearch Laboratories, West Grove, PA, USA) or peroxidase-AffiniPure F(ab')2 fragment donkey anti-rabbit IgG (1:10,000, polyclonal 711-036-152; Jackson ImmunoResearch Laboratories) for 1 h and rinsed three times for 5 s and once for 10 min in TBS-T. SuperSignal® West Femto Maximum Sensitivity Substrate (Thermo Fisher Scientific, Darmstadt, Germany) was used as a chemiluminescent detection substrate. Obtained signals were documented with the Fusion FX7 detection system (Vilber Lourmat, Eberhardzell, Germany).

### Imaging flow cytometry

IFCM analyses were performed on the AMNIS ImageStreamX Mark II Flow Cytometer (AMNIS/Luminex, Seattle, WA, USA), as described previously^[19-21]^. For staining, samples were incubated with CD9-PE (1:50, MEM61, Exbio) for 1 h at room temperature. All controls recommended by the MIFlowCyt-EV guidelines for flow cytometric EV analysis^[27]^ were performed, exactly as described previously^[20]^. After dilution with PBS, samples were measured using the integrated auto-sampler for 96-well U-bottom plates. Acquisition time was 5 min per well. Data were acquired at 60× magnification, low flow rate (0.3795 ± 0.0003 μL/min), and with removed beads option deactivated. Analysis was performed as described previously using IDEAS software version 6.2^[19]^.

### Nanoparticle tracking analysis

Samples were analyzed for particle size and concentration on a ZetaView™ PMX-120 BASIC platform (ParticleMetrix, Meerbusch, Germany). The machine was calibrated using a polystyrene bead standard (100 nm, Thermo Fisher Scientific). Samples were loaded and recorded at all 11 positions, with 5 repetitions. Further settings included: sensitivity 75, shutter 75, minimum brightness 20, minimum size 5, and maximum size 200. Each sample was measured three times. The videos were analyzed with the ZetaView Analyze program (Version 8.03.08.02); the median value (X_50_) for size was used for data analysis.

### Protein concentration analysis

The protein content of selected samples was determined using the bicinchoninic acid (BCA) protein assay kit (Pierce, Rockford, IL, USA). Protein analysis was performed in 96-well plates according to the recommendations of the manufacturer.

### Chloroform-methanol precipitation

The FFE fractions were too diluted for direct application to Western blots. Consequently, samples were concentrated by chloroform-methanol precipitation. Briefly, 800 μL methanol was added per 200 μL sample volume. After mixing, 200 μL chloroform and 600 μL ddH_2_0 were added. After repeated mixing, samples were centrifuged for 3 min at 14,000 *g*. Following protein concentration in the interphase, the upper aqueous phase was removed, and 800 μL methanol was added. Precipitated proteins were sedimented by centrifuging the samples for 5 min at 14,000 *g*. Supernatants were removed and pellets were air-dried for approximately 5 min. Dried pellets were resuspended in 40 μL non-reducing Laemmli buffer.

### Western blot

Western blot was performed as described previously^[22]^. Briefly, samples were separated on SDS-PAGE gels (12% tris-glycine/bis-acrylamide). Separated proteins were transferred to a polyvinylidene fluoride membrane (PVDF; Millipore, Darmstadt, Germany) by Wet Blot (Mini Trans-Blot® Cell, BioRad, Feldkirchen). TBS-T containing 5% (w/v) skim milk powder (Sigma) was used to block the PVDF membranes. The following antibodies were used to detect defined proteins: mouse anti-human CD9 antibody (TEA3, 1:1,000, 4 °C overnight, kindly provided by Francisco Sánchez-Madrid) and rabbit anti-human Syntenin antibody (1:1000, EPR8102, Abcam). All antibodies were diluted in TBS-T containing 5% (w/v) skim milk powder. Subsequently, membranes were washed four times (3× 10 s, 1× 10 min) in TBS-T and counterstained for 1 h at room temperature with Peroxidase-AffiniPure F(ab')2 Fragment donkey anti-mouse IgG (1:10,000; Jackson ImmunoResearch Laboratories) and peroxidase-AffiniPure F(ab')2 Fragment donkey anti-rabbit IgG (1:10,000; Jackson ImmunoResearch Laboratories). SuperSignal® West Femto Maximum Sensitivity Substrate (Thermo Fisher Scientific) was used as a chemiluminescent detection substrate.

### Transmission electron microscopy sample preparation

Transmission electron microscopy (TEM) was performed as described previously^[26]^. Briefly, 10 μL droplets of EV-containing samples were placed onto 200 mesh copper grids covered with carbon-coated formvar films (Plano GmbH, Wetzlar, Germany) for 5 min to allow the EVs to adhere to film surfaces. All following steps, contrasting, and fixation with 2% uranyl acetate were performed by placing the grids on droplets of different solutions. The incubations were conducted at room temperature.

Images were acquired using a JEOL JEM 1400Plus (JEOL Ltd., Tokyo, Japan), operating at 120 kV and equipped with a 4096 × 4096 pixels CMOS camera (TemCam-F416, TVIPS, Gauting, Germany). Image acquisition software EMMENU (Version 4.09.83) was used for taking 16-bit images. Image post-processing was carried out using ImageJ (Version 1.52b).

## RESULTS

### FFE allows separation of protein and EV contents from PEG prepared MSC-EV samples

Aiming to test the suitability of FFE for the fractioning of complex EV-containing samples, we started our experimental series with MSC-EV samples routinely prepared in our lab using a combined PEG/UC preparation method^[14,22]^. First, 133 μL of a well-characterized MSC-EV preparation (MSC-EV 31.2) that had been administered to ischemic stroke mice^[26]^ were fractioned within 6 min with a free-flow interval zone electrophoresis protocol using five different pH zones (pH 4.8, 5.4, 6.4, 7.4, and 8.4) flanked by anode and cathode stabilization buffers (pH 8.3) [Figure 2A]. In total, 96 different fractions (1230 μL each) were collected from the separation area, including the regions with the anode and cathode stabilization buffers. Analyses of the protein content (UV280 nm) of different fractions revealed that most of the protein was recovered in Fractions 44-80 (absorbance at 280 nm > 1500) [Figure 2B]. To identify the EV-containing fractions, a dot plot immunostaining was performed. Coupled with the usage of hPL supplemented media, the most abundant EV population in our MSC-EV samples is the CD9^+^CD81^-^ EV population^[21]^, expectedly also being positive for the EV marker Syntenin^[17]^. Consequently, we decided to focus on anti-CD9 and anti-Syntenin analyses. Accordingly, 200 μL of each obtained FFE fraction was applied and probed with a mixture of anti-CD9 and anti-Syntenin antibodies. Positive signals were detected in Fractions 35-37, which were derived from the pH 4.8 zone, adjacent to the anode stabilization buffer [Figure 2C]. Since these fractions had a rather low protein content, we concluded that fractioning by FFE separates protein impurities in EV preparations from their EV content.

### FFE allows separation of CD9^+^ objects and protein contents from MSC-CMs

Intending to use FFE for the preparation of EVs from CMs, we decided to next prepare EVs from MSC-CMs. An MSC-CM (CM1) was used for this experiment that had been cleared from cells and larger particles and had a protein concentration of 4.39 mg/mL, according to NTA average particle sizes of 116.2 ± 8.1 nm and a particle concentration of 2.50 × 10^10^ particles/mL. According to IFMC, the content of CD9^+^ objects was 3.14 × 10^8^ objects/mL MSC-CM, resulting in purity indexes of 5.69 × 10^9^ particles/mg protein and 7.14 × 10^7^ CD9^+^ objects/mg protein, respectively [Table 1].

**Table 1:** Protein, IFCM and NTA data of MSC-CM

	**Prot conc**	**CD9^+^ obj conc**	**Obj purity index**	**Total CD9^+^ obj**	**Particle size**	**Particle conc**	**Part purity index**	**Total particles**
**Method**	BCA	IFCM	IFCM/BCA	IFCM × Vol	NTA	NTA	NTA/BCA	NTA × Vol
	[mg/ml]	[obj/mL]	[obj/mg]	[obj/3.125 mL]	[nm]	[particle/mL]	[particle/mg]	[part/3.125 mL]
**CM1**	4.39	3.14E+08	7.14E+07	9.81E+08	115.1	2.50E+10	5.69E+09	7.81E+10
**CM2**	4.76	1.85E+08	3.87E+07	5.77E+08	113.0	2.20E+10	4.62E+09	6.88E+10
**CM3**	4.64	2.84E+08	6.12E+07	8.88E+08	118.0	3.10E+10	6.68E+09	9.69E+10
**CM4**	4.57	2.36E+08	5.17E+07	7.39E+08	115.1	2.20E+10	4.81E+09	6.88E+10
**CM5**	4.80	2.46E+08	5.13E+07	7.70E+08	117.3	2.60E+10	5.41E+09	8.13E+10

IFCM: Imaging flow cytometry; NTA: nanoparticle tracking analysis; BCA: bicinchoninic acid.

To decrease the processing time, the separation protocol was optimized and simplified. Instead of five buffers with different pH values, three different buffers were chosen for the separation area: a high conductivity buffer with pH 4.5, a low conductivity buffer of pH 4.5, and a buffer with pH 7.1. These buffers were flanked by anode and cathode stabilization buffers with pH 7.3; the separation area was reduced to half of the separation chamber [Figure 3A]. Applying the optimized protocol, 3.125 mL of MSC-CM were processed in 25 min. In total, 48 different fractions were collected, each with a volume of 1.8 mL. In addition, for the spectroscopic evaluation of the stability of the separation process, 150 mL aliquots of all fractions were collected before and after the scaled sampling process. The obtained pherograms revealed that most of the protein content was recovered in Fractions 7-8 and 23-27 [Figure 3B]. Upon testing 200 μL of each of the obtained samples in the dot plot procedure, expectedly, we failed to detect any EV proteins due to the much lower EV concentration within the starting material. Since, according to our experience, IFCM is more sensitive than Western or Dot blot, we decided to analyze relevant fractions by IFCM. Preliminary IFCM analyses performed during the FFE protocol establishment period qualified anti-CD9 labeling as a robust and sufficiently sensitive method for the identification of EV-containing samples. These analyses demonstrated that CD9^+^ objects, assumedly CD9^+^ EVs, were exclusively recovered in up to three fractions, all within the range of Fractions 7-10. Consequently, following the processing of MSC-CM with the optimized protocol, we focused our anti-CD9 IFCM analyses on Fractions 6-11. In our proof-of-principle run, Fractions 6-8, 10, and 11 hardly contained any CD9^+^ objects. In contrast, Fraction 9 contained more than 3.12 × 10^7^ CD9^+^ objects per mL [Figure 3C, Supplementary Figure 3, Table 2]. As before, the fraction containing most CD9^+^ objects had a relatively low protein content, resulting in a purity of 9.24 × 10^7^ objects/mg protein [Table 2]. These results imply that FFE might also be a suitable method for preparing EVs from MSC-CMs to fair purities.

**Table 2:** Protein, IFCM and NTA data of obtained FFE Fractions 7-10

	**FFE-Fraction**	**Prot conc**	**CD9^+^ obj conc**	**Obj purity index**	**Total CD9^+^ obj**	**Particle size**	**Particle conc**	**Part purity index**	**Total particles**	**Recovery**	**Recovery**
**Method**		BCA	IFCM	IFCM/BCA	IFCM × Vol	NTA	NTA	NTA/BCA	NTA × Vol	**CD9^+^ obj**	**Particles**
		[mg/ml]	[obj/mL]	[obj/mg]	[obj/1.8 mL]	[nm]	[particle/mL]	[particle/mg]	[part/1.8 mL]		
**EV (CM1)**	7	0.046	1.22E+05	2.63E+06	2.19E+05	127.3	9.80E+08	2.12E+10	1.76E+09	0.02%	2.26%
	8	0.209	0.00E+00	0.00E+00	0.00E+00	146.6	2.40E+09	1.15E+10	4.32E+09	0.00%	5.53%
	**9**	**0.337**	**3.12E+07**	**9.24E+07**	**5.61E+07**	**131.4**	**1.50E+10**	**4.45E+10**	**2.70E+10**	**5.72%**	**34.56%**
	10	0.118	6.36E+03	5.40E+04	1.14E+04	134.8	4.00E+09	3.40E+10	7.20E+09	0.00%	9.22%
**EV (CM2)**	7	0.152	9.94E+04	6.55E+05	1.79E+05	134.9	5.20E+08	3.43E+09	9.36E+08	0.03%	1.36%
	8	0.186	0.00E+00	0.00E+00	0.00E+00	133.8	1.20E+09	6.47E+09	2.16E+09	0.00%	3.14%
	**9**	**0.377**	**1.19E+07**	**3.15E+07**	**2.14E+07**	**117.2**	**7.90E+09**	**2.10E+10**	**1.42E+10**	**3.71%**	**20.68%**
	10	0.270	3.22E+06	1.19E+07	5.80E+06	113.3	4.40E+09	1.63E+10	7.92E+09	1.01%	11.52%
**EV (CM3)**	7	0.176	0.00E+00	0.00E+00	0.00E+00	123.9	6.40E+08	3.63E+09	1.15E+09	0.00%	1.19%
	8	0.203	0.00E+00	0.00E+00	0.00E+00	116.0	6.10E+08	3.00E+09	1.10E+09	0.00%	1.13%
	**9**	**0.360**	**2.27E+07**	**6.31E+07**	**4.09E+07**	**110.1**	**1.40E+10**	**3.89E+10**	**2.52E+10**	**4.61%**	**26.01%**
	10	0.102	2.73E+06	2.67E+07	4.91E+06	135.8	5.10E+09	5.00E+10	9.18E+09	0.55%	9.48%
**EV (CM4)**	7	0.155	4.45E+04	2.87E+05	8.01E+04	139.4	3.00E+08	1.94E+09	5.40E+08	0.01%	0.79%
	8	0.180	1.06E+03	5.88E+03	1.90E+03	144.8	1.50E+09	8.34E+09	2.70E+09	0.00%	3.93%
	**9**	**0.417**	**1.37E+07**	**3.29E+07**	**2.47E+07**	**116.7**	**9.20E+09**	**2.21E+10**	**1.66E+10**	**3.34%**	**24.09%**
	10	0.214	3.87E+06	1.81E+07	6.97E+06	119.0	4.00E+09	1.87E+10	7.20E+09	0.94%	10.47%
**EV (CM5)**	7	0.193	4.23E+04	2.20E+05	7.61E+04	170.6	3.80E+08	1.97E+09	6.84E+08	0.01%	0.84%
	8	0.247	0.00E+00	0.00E+00	0.00E+00	148.8	1.90E+09	7.68E+09	3.42E+09	0.00%	4.21%
	**9**	**0.394**	**1.74E+07**	**4.42E+07**	**3.13E+07**	**133.1**	**1.20E+10**	**3.05E+10**	**2.16E+10**	**4.07%**	**26.58%**
	10	0.165	2.52E+06	1.53E+07	4.54E+06	140.1	5.10E+09	3.09E+10	9.18E+09	0.59%	11.30%

IFCM: Imaging flow cytometry; NTA: nanoparticle tracking analysis; BCA: bicinchoninic acid; FEE: free flow electrophoresis; EV: extracellular vesicle.

### FFE allows preparation of bona fide EVs 

**Figure 4:**
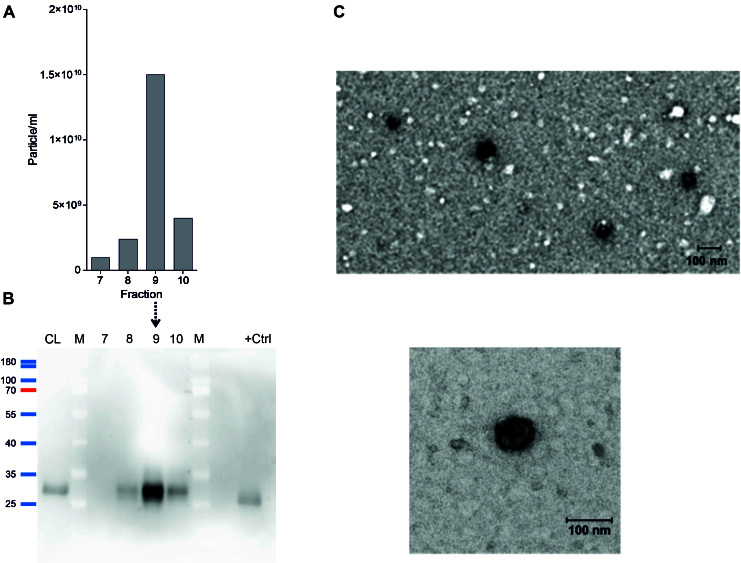
Free flow electrophoresis (FEE) allows preparation of *bona fide *extracellular vesicles (EVs). FFE fractions considered to contain the majority of EVs were analyzed by nanoparticle tracking analysis (NTA), Western blot (WB), and Transmission electron microscopy (TEM). (A) The results of NTA analyses depicted as particles per mL and mean particle size (131.4 nm) of Fractions 7-10 of the FFE fractioned MSC-CM (for details, see CM1 in Table 1). (B) Anti-CD9 Western blot of the same fractions depicted in (A). (C) TEM images of detectable Fraction 9 components following uranylacetate fixation: (top) 10,000× (1.189 nm/px) magnification (120 kV); and (bottom) 50,000× (0.240 nm/px) magnification.

Although our previous results qualified IFCM as a second-generation EV analysis method for the detection and characterization of EVs including small EVs at the single-EV level^[17,19-21]^, we also characterized Fractions 7-10 by NTA and Western blot (WB) and determined their protein content in addition to the spectral analysis by BCA [Tables 1 and 2]. Consistent with the number of CD9^+^ objects, the highest particle counts, 1.5 × 10^10^ particles per mL as determined by NTA, were found in Fraction 9, which as a consequence thereof was also analyzed by TEM. Fractions 7, 8, and 10 contained clearly fewer particles [Table 2]. The average sizes of the recorded particles were 127.3 ± 7.9 (Fraction 7), 146.6 ± 13.9 (Fraction 8), 131.4 ± 6.2 (Fraction 9), and 134.8 ± 8.3 nm (Fraction 10) [Figure 4A]. Due to the low protein concentration of the obtained FFE fractions, the protein content was concentrated by chloroform-methanol precipitation (200 μL aliquots each) and analyzed by WB. Signals for CD9 were obtained within the lanes of Fractions 8-10, with the strongest band in the lane of Fraction 9 [Figure 4B]. Unfortunately, most likely due to the low protein concentration of the prepared samples, other EV specific proteins were not successfully detected in Western blots (data not shown). TEM analyses revealed objects with a vesicular appearance in Fraction 9 [Figure 4C]. Altogether, these data imply that FFE Fraction 9 contains *bona fide* EVs. Thus, FFE can also quickly prepare EVs from cell culture supernatants.

### FFE-is highly reproducible and robust for protein fractioning

After gaining evidence that FFE allows the preparation of *bona fide* EVs from MSC-CM, we explored the reproducibility of the method. To this end, in addition to the previous MSC-CM (CM1), we included CMs from four additional MSC stocks (CM2-5). All MSC-CMs revealed protein concentrations between 4.3 and 4.8 mg/mL, their average particle concentration varied between 2.2 and 3.1 × 10^10^ particles/mL, and their number of CD9^+^ objects between 1.85 and 3.14 × 10^8^ per mL [Table 1]. The purity indexes varied between 4.64 and 6.68 × 10^9^ particle/mg protein and between 3.87 and 7.14 × 10^7^ CD9^+^ objects/mg protein [Table 1].

**Figure 5:**
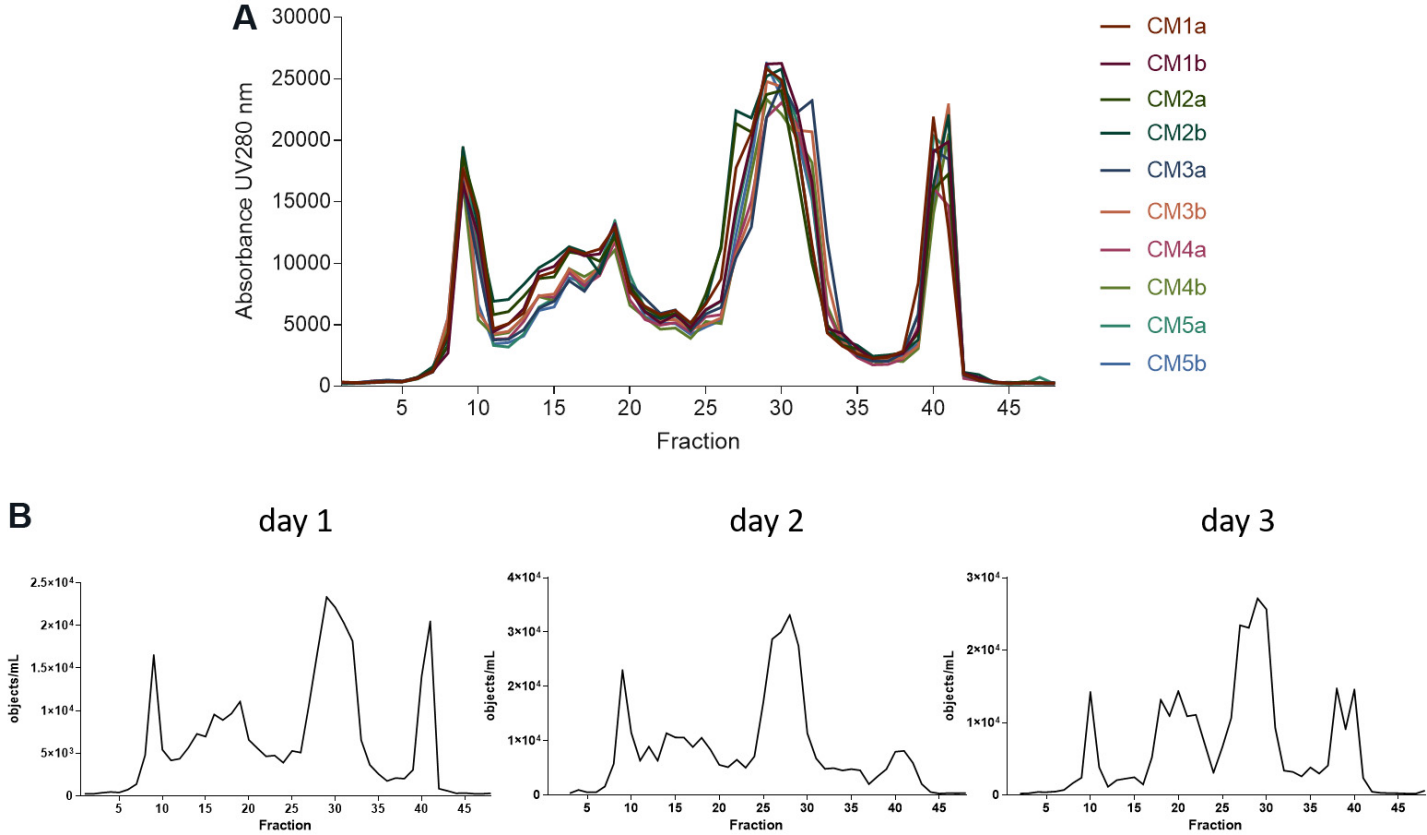
Free flow electrophoresis (FEE) fractioning is highly reproducible and robust. (A) Pherograms of obtained fractions of five different MSC-CMs (CM1-5) all of which were processed two independent times on the same day (a, b). (B) Pherograms of fractions obtained of MSC-CM1 that were fractioned on three different days. FFE conditions were applied as indicated in Figure 3.

Applying the former FFE protocol, fractioned aliquots of the five different MSC-CMs were fractioned on three different days, with two independent runs per MSC-CMs and day. As an initial analysis, the protein content of all 48 obtained fractions of each individual run was determined spectroscopically, measuring the light absorption of each individual fraction at 280 nm. Upon comparing the obtained pherograms, we observed a high degree of reproducibility of the method. All pherograms obtained from MSC-CMs that had been fractioned on the same day were almost identical [Figure 5A], indicating extreme robustness and reproducibility of the process, at least for intra-day performances. Pherograms obtained from identical MSC-CMs that were fractioned on different days were very similar to each other as well, but they showed some minor differences [Figure 5B]. According to our experience, these slight differences in the day-to-day performance are due to minor, unavoidable variations in setting up the FFE aperture. Overall, however, in terms of protein fractioning, our results demonstrate FFE is highly reproducible and robust.

### FFE reproducible allows preparation of EVs from MSC-CM

**Figure 6:**
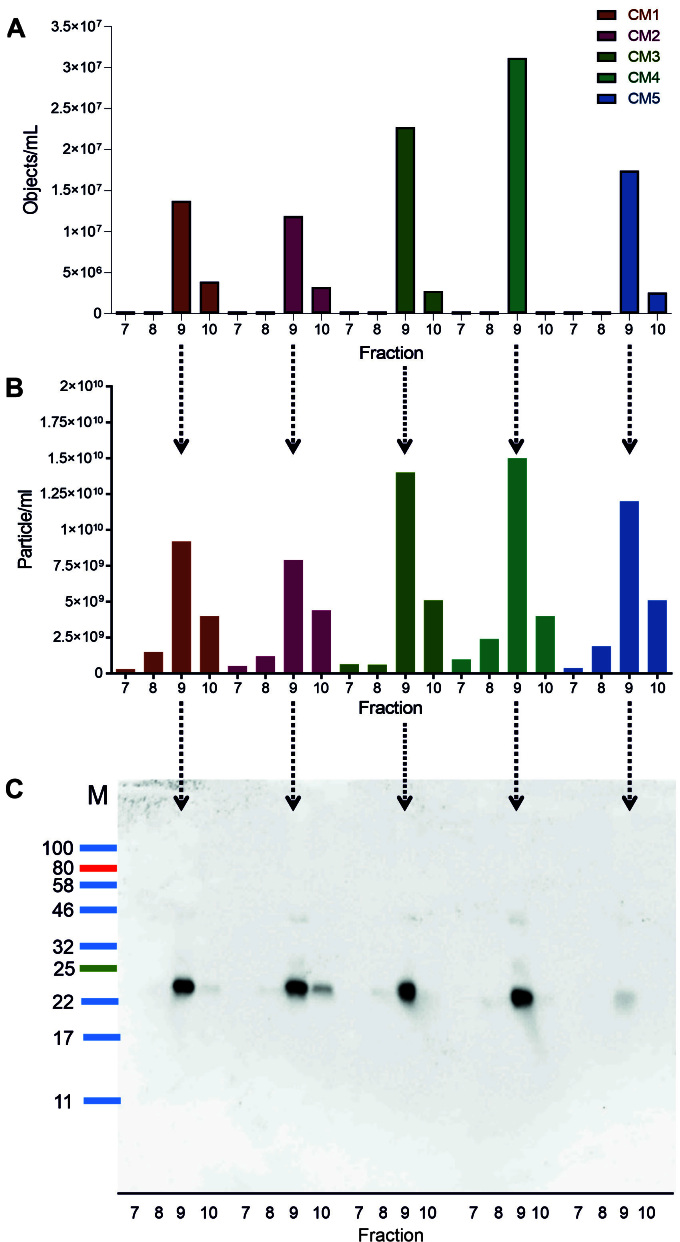
Free flow electrophoresis (FEE) is reproducible, allowing preparation of extracellular vesicles (EVs) from MSC-CMs. Five MSC-CMs were fractioned on the same day by FFE applying the protocol indicated in Figure 4. To evaluate the reproducibility of the EV preparation, obtained Fractions 7-10 were analyzed: (A) following anti-CD9 labeling by imaging flow cytometry; (B) by nanoparticle tracking analysis; and (C) following chloroform-methanol precipitation by anti-CD9 Western blot (exposure time 270 s).

After learning FFE reproducibly fractions different MSC-CMs regarding their protein concentration, we next investigated Fractions 7-10 for their CD9^+^ object and particle content by IFCM and NTA, respectively. Furthermore, samples of these fractions were investigated by anti-CD9 Western blots. The IFCM data reveal that consistently the majorities of CD9^+^ objects were recovered in Fraction 9 in all cases, with the exception of MSC-CM4, where minor proportions of CD9^+^ objects were also recovered in Fraction 10 but never in Fraction 7 or 8 [Figure 6A, Table 2]. In good agreement, NTA also detected the highest particle content in all Fraction 9 samples (between 7.9 × 10^9^ and 1.2 × 10^10^ particle/mL and 1.19 and 3.12 × 10^7^ CD9^+^ objects/mL) followed by Fraction 10 in all cases [Figure 6B, Table 2]. According to the purity indices expressed as particles per mg protein, Fraction 9 contained between 4.54 and 7.82 times more particles per mg protein than the original CMs. The average sizes of the recorded particles in Fraction 9 were between 110.1 and 133.1 nm [Table 2]. Following chloroform-methanol precipitation of the samples, the data were further substantiated by results of Western blots, which also showed the highest anti-CD9 signal intensities in all Fraction 9 samples [Figure 6C]. Despite the fact that, assumedly due to issues in chloroform-methanol precipitation, the CD9 band intensity of the Fraction 9 of MSC-CM5 is somehow weaker than that of the other Fraction 9 samples, altogether, the data demonstrate that CD9^+^ EVs can specifically be recovered in Fraction 9. Thus, FFE allows reproducible enrichment of EVs from MSC-CMs.

## DISCUSSION

Small EVs, especially exosomes, were discovered almost 40 years ago, and the first report on their functional impacts was reported in 1996^[28-30]^. However, EV preparation remains challenging, and, to our best knowledge, no technologies have been described allowing the preparation of reasonably pure EVs in short time intervals. Here, we evaluated the suitability of FFE for the fractioning of EVs from a preclinically tested MSC-EV sample and the preparation from MSC-CMs. We demonstrated that CD9^+^ EVs from given EV preparations or MSC-CMs are very reproducibly recovered in discrete fractions, specifically in 3 out of 96 or 48 fractions, respectively, while most of the protein is recovered in other fractions. Thus, FFE allows quick and reproducible separation of EVs from a huge proportion of other molecules and compounds included in the original EV-containing samples. Depending on the EV starting concentration and the experimental needs, a continuous separation procedure can be performed, as demonstrated here by preparing EVs from more than 3 mL of MSC-CMs within 25 min. Since the application zone is much broader than that of each collected fraction and electrophoresis results in isoelectric focusing, analytes can be slightly concentrated compared to the starting samples. However, even though FFE can be used for EV preparation, the preparation remains low scale and is not necessarily quantitative. For example, after the application of samples, the performance of the separation process should be stabilized before beginning with the collection of the different fractions. Comparably, the collection period should be finished before the last sample components reach the collector unit, resulting in the additional loss of sample material. However, FFE provides several new and beneficial options for EV research. The pherograms of the obtained fractions, for example, reveal information about the complexity of the initially applied EV samples. As long as proteins are recovered in other non-EV FFE fractions, they can be considered as byproducts or impurities. In terms of EV-based therapeutics, it is important to understand that byproducts may contribute to the therapeutic effect of the EV product and can be tolerated as long as they neither negatively affect the product’s function nor cause any side effects and as long as the product assembly is reproducible in independent batches of the same EV-product type^[31]^. To demonstrate the reproducibility of the molecular composition of obtained EV samples, we previously discussed the need for a fingerprinting method for such products, especially when their clinical application is considered^[32]^. Due to its high reproducibility, we understand FFE as a very potent method for generating reliable and reproducible fingerprints of obtained EV products and envision its huge potential for quality control of EV-based therapeutics.

Furthermore, combined with imaging flow cytometry, which can be performed in an automatized manner in the 96-well format and which we optimized for single EV analyses^[19-21]^, EV-containing fractions can quickly be identified and used for different down-stream analyses, as exemplarily shown for NTA, Western blot, and TEM.

As exemplified in this manuscript, different separation profiles can be applied for the separation of EVs from proteins and other sample components. We may not have established the perfect EV preparation protocol for MSC-EV or MSC-CM samples here. Indeed, proteomic profiling of obtained EV-containing fractions still recovered impurities in our CD9^+^ EV fractions (data not shown). However, by using different separation buffers, the separation capabilities can be significantly influenced and possibly improved much further, such that FFE will finally also allow separation of different EV subtypes. Indeed, in ongoing projects, in which we are investigating the usefulness of FFE for the preparative separation of EVs from other plasma sample components, we can recover different EV subtypes in different FFE fractions and are currently unraveling this EV heterogeneity in more detail (data not shown). This series of preliminary results implies that different EV types differ regarding their ionic strengths or pIs, respectively, likely providing another level of complexity to the EV field.

In summary, we demonstrated that FFE is a fast and efficient method to separate EVs from a huge proportion of other sample components included in different EV-containing liquids. Our results reflect the high reproducibility of the FFE-based sample separation and qualify FFE as an ideal device for the molecular fingerprinting of obtained EV products. 

## DECLARATIONS

### Availability of data and materials

Additional data is provided within the supplements.

### Copyright

© The Author(s) 2022.
