# Supplementary material for: Free flow electrophoresis allows quick and reproducible preparation of extracellular vesicles from conditioned cell culture media
Source: Extracell Vesicles Circ Nucl Acids. 2022 Mar 16;3(1):31–48. doi: 10.20517/evcna.2021.26 (PMC11651122; doi:10.20517/evcna.2021.26)
Supplement: Supplementary file 1 [file evcna-3-1-31-SupplementaryMaterials.pdf]

Set up

- **Buffer set up:** mix buffer components and adjust pH and conductivity
- **System set up:** loading the device with buffers; remove air bubbles, check fluid leaks (inlets and outlets)
- **Stripe test:** check for the stable laminar flow
- **Performance test**: check for the electrophoretic separation capability

Separation

- **Sample application** in proximity of the cathode
- **(pre-scaled) sampling** into a microtiter plate (150-200 µL per well; ≈3 min) *if scaled sample volumes are* required
- **scaled sampling** into a deep well plate (up to 1.8 mL per well; ≈ 25 min)
- **post-scaled sampling** into a microtiter plate (150-200 µL per well; ≈3 min)

Analysis

- **Spectral analyses and generation of pherograms**
- **Evaluation of the process stability** (comparison of the pherograms obtained from the pre- and post-scaled sampling)
- **Identification of EV containing samples** (dot blot or IFCM analyses)
- **Validation of the EV nature** (WB, NTA, TEM).
- **Optional**: down-stream methods (e.g. proteomics)

Supplementary Figure 1. Schematic-workflow-new-1185.

A

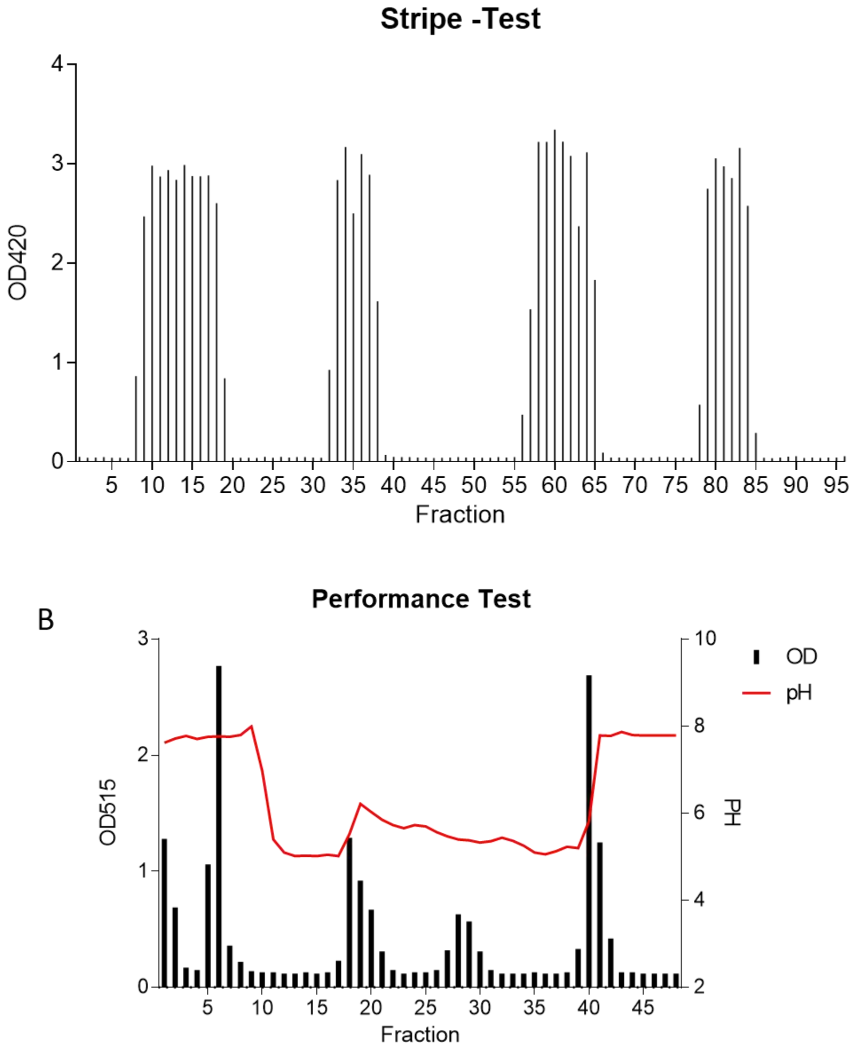

B

Supplementary Figure 2. Stripe-and-Performance-Test-3815.

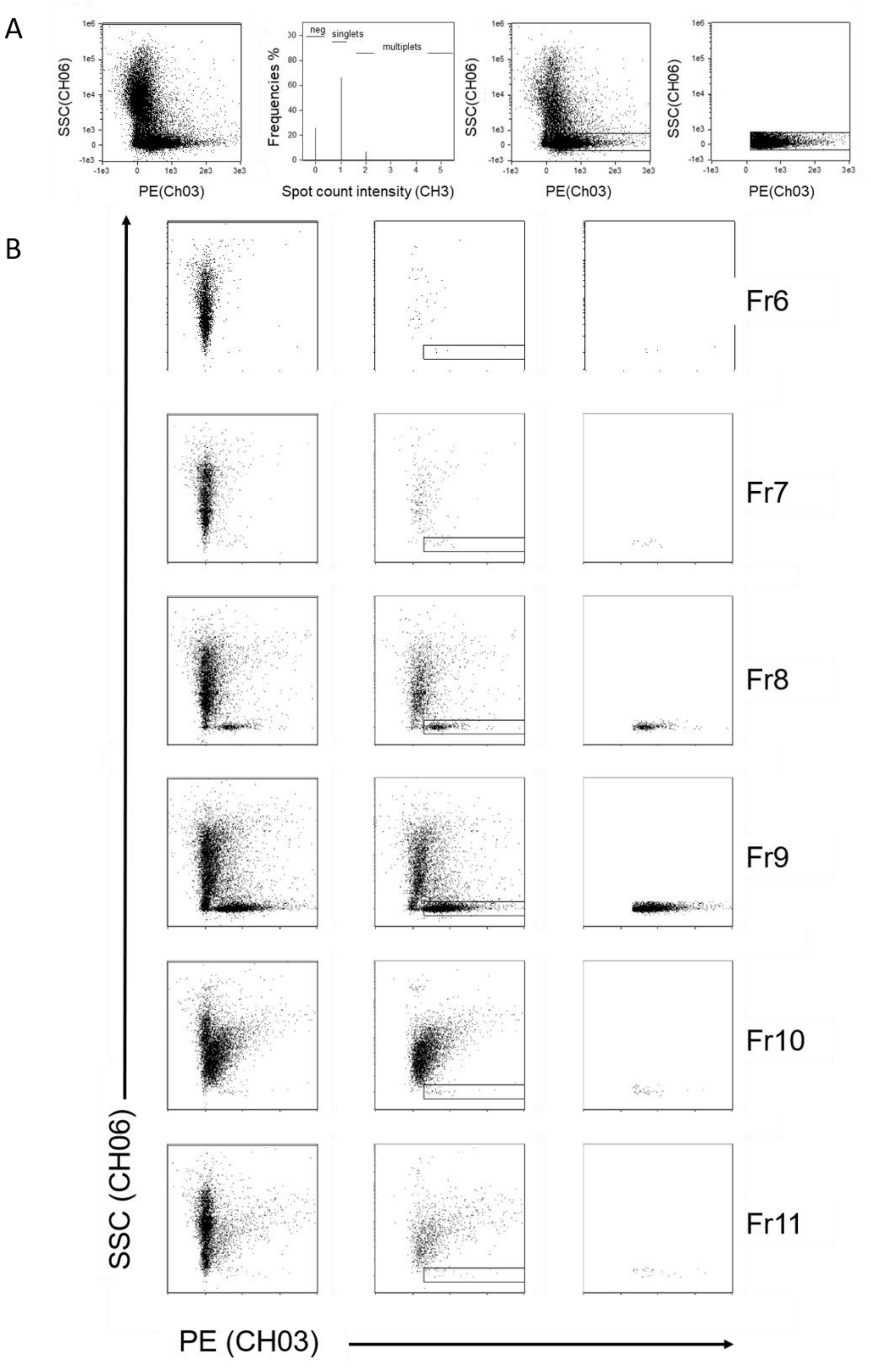

Supplementary Figure 3. Gating+IFCM-1720.
